# *In Situ* Persistence and Migration of Biochar Carbon and Its Impact on Native Carbon Emission in Contrasting Soils under Managed Temperate Pastures

**DOI:** 10.1371/journal.pone.0141560

**Published:** 2015-10-28

**Authors:** Bhupinder Pal Singh, Yunying Fang, Mark Boersma, Damian Collins, Lukas Van Zwieten, Lynne M Macdonald

**Affiliations:** 1 NSW Department of Primary Industries, Elizabeth Macarthur Agricultural Institute, Menangle, NSW, 2568, Australia; 2 University of Tasmania, Tasmanian Institute of Agriculture, Burnie, Tasmania, 7320, Australia; 3 NSW Department of Primary Industries, Wollongbar Primary Industries Institute, Wollongbar, NSW, 2477, Australia; 4 CSIRO Agriculture, Glen Osmond, South Australia, 5064, Australia; Tennessee State University, UNITED STATES

## Abstract

Pyrogenic carbon (PyC) is an important component of the global soil carbon (C) pool, but its fate, persistence, and loss dynamics in contrasting soils and environments under planted field conditions are poorly understood. To fill this knowledge gap, a ^13^C-labelled biochar, as a surrogate material for PyC, produced from *Eucalyptus saligna* by slow pyrolysis (450°C; δ^13^C -36.7‰) was surface (0−10 cm) applied in C_3_ dominated temperate pasture systems across Arenosol, Cambisol and Ferralsol. The results show a low proportion of the applied biochar-C mineralised over 12 months in a relatively clay- and C-poor Arenosol (i.e., 2.0% loss via mineralisation), followed by a clay- and C-rich Cambisol (4.6%), and clay-, C- and earthworm-rich Ferralsol (7.0%). The biochar-C mean residence time (MRT), estimated by different models, varied between 44−1079 (Arenosol), 18−172 (Cambisol), and 11−29 (Ferralsol) years, with the shorter MRT estimated by a one-pool exponential and the longer MRT by an infinite-pool power or a two-pool exponential model. The two-pool model was best fitted to biochar-C mineralisation. The biochar-C recovery in the 12−30 cm soil layer varied from between 1.2% (Arenosol), 2.5−2.7% (Cambisol) and 13.8−15.7% (Ferralsol) of the applied biochar-C after 8−12 months. There was a further migration of biochar-C below the 50-cm depth in the Arenosol, as the combined biochar-C recovery in the mineralised pool and soil profile (up to 30 or 50 cm) was 82%, in contrast to 101% in the Cambisol and 104% in the Ferralsol after 12 months. These results indicate that the downward migration of biochar-C was greatest in the Arenosol (*cf*. Cambisol and Ferralsol). Cumulative CO_2_-C emission from native soil-plant sources was lower (*p* <0.10) in the biochar-amended *vs*. non-amended Ferralsol. This field-based study shows that the downward migration of biochar-C exceeded its loss *via* mineralisation in the Arenosol and Ferralsol, but not in the Cambisol. It is thus important to understand biochar-soil interactions to maximise long-term biochar C sequestration potential in planted soil systems.

## Introduction

Pyrolysed carbon (PyC) is produced naturally during fires following incomplete combustion of biomass [1] and is an important but poorly understood pool of the global carbon (C) cycle. Because of its relatively recalcitrant nature, some PyC is preserved in soil and sediments for several decades to millennia [2, 3] and constitutes up to 45% of organic C in soil [4–7]. PyC can be lost from soil via mineralisation (albeit at a much slower rate than C in parent biomass), dissolution and leaching, and wind/water erosion [6, 8]. Understanding the fate, persistence and reaction of PyC in soil is crucial for constraining C models to accurately predict PyC sequestration potential and evaluate its contribution to the global C budget. Biochar is a form of PyC produced intentionally *via* pyrolysis under controlled conditions from plant biomass or bio-waste. Biochar may be a good surrogate for natural PyC as it has similar, or indeed greater, C sequestration characteristics [9, 10]. Thus, biochar could also improve our understanding of the fate and persistence of PyC and its influence of native plant-soil organic C in terrestrial ecosystems.

The global interest in using biochar for organic waste management, C management, and as an amendment to improve soil health, soil C, and agricultural productivity has risen rapidly over the last decade [10, 11]. Additionally, because of its ability to improve nutrient retention and use efficiency, reduce nutrient leaching, and mitigate GHG emissions [12–15], biochar application has been recommended for ‘high input’ temperate pasture systems to achieve the above-mentioned multiple benefits [16]. Temperate pastures cover 1.25 × 10^9^ ha worldwide and are an important sink of soil C, representing approximately 12% of the soil organic C globally [17]. The C storage potential, GHG mitigation and plant productivity of the temperate pasture systems could be enhanced through improved management practices [18, 19], including through the application of biochar [20]. Several laboratory [21–26] and some field studies [27, 28] indicate that biochar is a highly persistent organic material in soil. Due to its high C stability, it has been proposed that biochar could be an effective tool as a long-term C sink in terrestrial systems to mitigate climate change [29].

Using C isotope-distinct biochars (relative to native soil C), several laboratory incubation studies have quantified biochar-C persistence under controlled conditions [21, 22, 24, 30, 31]. It has been shown that biochar persistence in soil is a function of soil characteristics (*e*.*g*. clay mineralogy, native soil C content, texture, microbial activity [22, 32, 33]); plant residue input [34]; and environmental factors (*e*.*g*. temperature, moisture [7, 35, 36]). Thus far, there have been only limited field-based *vs*. laboratory-based studies, and particularly field studies assessing the fate and persistence of biochar across different soil types and environmental conditions are lacking. In a field study including plant C input, Major et al. [27] reported 3% loss of a naturally ^13^C-depleted woody biochar (i.e. C_3_-biochar *vs*. C_4_-soil organic matter) *via* mineralisation over a two-year period in an Oxisol. In another field study without the presence of plant roots that employed a highly ^13^C-enriched biochar (PyC), Singh et al. [28] and Maestrini et al. [37] quantified less than 1% PyC was mineralised in a Cambisol over 10−12 months. In a recent field-based study, using the same biochar as in the present study, Weng et al. [38] showed that the presence or absence of plants did not alter biochar-C mineralisation but the presence of plants decreased native soil organic C (SOC) mineralisation in a sesquioxide-rich Ferralsol. This is likely due to rapid development of organo-biochar-mineral interactions and stabilisation of SOC in aggregates [39, 40]. Our current understanding of how soil types interact to influence biochar-C mineralisation is limited under planted field conditions. Hence, the sequestration potential of PyC in terrestrial ecosystems remains highly uncertain [41].

In the field, biochar-C or PyC can also be lost *via* lateral movement across the landscape or downward migration in the soil profile [28, 42–44]. Surface erosion by wind and water, and subsurface leaching and infiltration, have been suggested as main pathways of PyC or biochar-C export from the terrestrial to marine systems [45–47]. The field study by Major et al. [27] reported that 20–53% of biochar-C was lost by soil surface erosion with a 2% slope in a sandy Oxisol. Major et al. [27] also reports a simultaneous migration of up to 0.02% of applied biochar-C from 0.1 to 0.3 m in particulate and dissolved organic C forms over a two-year period. In a clay loam Cambisol, Singh et al. [28] found a migration of 3–4% of the surface applied (top 2 cm) biochar-C to 5−15 cm depth, while a negligible loss of this biochar occurred via leaching as dissolved organic C [37]. On the other hand, Jaffé *et al*. (2013) reports an annual global export 26.5 ± 1.8 Tg of PyC from the land to the ocean, which represents about 10% of the total riverine DOC flux [47]. The downward migration of biochar-C can be facilitated by the coarse-textured nature of soil [47], or *via* bioturbation [27, 47] particularly in soil systems where macrobiota activity is high. There is however a lack of information on the extent of downward migration of biochar-C or Py-C in different soil types and/or environments under planted field conditions. This information is crucial for evaluating the overall persistence of biochar or PyC in natural environment.

This field-based study aimed to provide insights into the persistence, fate and mobility of biochar or PyC and its impact of native C sources in contrasting soil systems with varying SOC content, clay content, and earthworm activity under ‘high input’ pasture systems. Thus, based on the findings reported in the current literature, we hypothesised for this field-based study that (i) biochar-C mineralisation would be higher in the SOC-rich soil (Cambisol or Ferralsol) that simultaneously supported greater plant productivity and/or earthworm activity than in the SOC-poor soil (Arenosol) with limited plant productivity or no earthworm activity; (ii) biochar will decrease native C emission from the relatively clay- and C-rich soil (Ferralsol) *vs*. a clay- and C-poor soil (Arenosol), possibly due to negative priming of SOC; and (iii) the coarser-textured Arenosol or greater earthworm activity in the Ferralsol (combined with relatively high rainfall) will favour downward migration of biochar through the soil profile. A ^13^C labelled *Eucalyptus saligna* biochar was used to quantify biochar-C mineralisation, its impact on native C emissions, and its downward migration in three soils under temperate pastures.

## Materials and Methods

### Experimental field sites

The experimental field sites were located in (a) Cobbitty, New South Wales (NSW), Australia and (b) Elliot, Tasmania (TAS), Australia. The Cobbitty site was under a warmer and drier temperate environment, whereas the Elliot site was under a colder and wetter temperate environment. The field site in Cobbitty was previously under a mix of arable and fallow land uses during the experimental period. The field site in Elliott was previously under a long term dairy pasture. The soils at the Cobbitty field site are classified as Tenosol (-34.02140°, 150.66227°) and Dermosol (-34.02340°, 150.66350°) and the soil at the Elliott site (-41.08110°, 145.77035°) is classified as Ferrosol as per the Australian Soil Classification [48]. The Tenosol, Dermosol and Ferrosol at the selected sites are equivalent to Arenosol, Cambisol and Ferralsol, respectively, as per the World Reference Base [49]; or Inceptisol, Alfisol and Oxisol, respectively, as per the USDA Soil Classification [50]. Permissions were granted for establishing and accessing the field micro-plot trials by the University of Sydney for the Cobbitty site and the University of Tasmania for the Elliott site. The field sites did not involve endangered or protected species.

### Environmental factors

Soil temperature in the micro-plots (see below) was monitored every two or four hours at 5 cm below the surface using i-buttons (Maxim Integrated, San Jose, California, USA). Volumetric moisture content at 10 cm was monitored manually on the day of gas sampling by a soil water sensor (HydroSence II).

### Experimental design and establishment of micro-plots

The experimental areas were established as strips of approximately 13 m length and 7 m width (one strip for each soil type), which were power harrowed to 10 to 15 cm depth. The strips were sown (broadcast seeding) with mixed C_3_ pastures, as appropriate practices for the regional areas. The experimental strips on the Arenosol and Cambisol (Cobbitty site) were fertilised with 110 kg N ha^-1^ as urea at sowing, followed by its reapplication (55 kg N ha^-1^) three months after sowing. The experimental strip on the Ferralsol (Elliott site) was fertilised with 46 kg N ha^-1^ as urea at sowing, and after pasture establishment, urea was reapplied every 20 to 30 days at the equivalent of 1 kg N ha^-1^ day^-1^ (a common practice for a high input dairy pasture in the region in Tasmania). The sowing rate at the Cobbitty site was 15 kg ha^-1^ of mixed pasture seeds, including lucerne, phalaris, fescue, prairie grass, subterranean clover (2 vars.), medic, arrowleaf clover, white clover, strawberry clover and bladder clover. This mixed pasture sward is typical of the temperate region in NSW and ensures good persistence of the plant cover to variable wet/dry annual conditions [51]. The Elliott site was seeded with 30 kg ryegrass ha^-1^ while white clover re-established in the plots via self-seeding. The strips at the field sites were irrigated periodically as needed.

For all the sites, a randomised block design was used to divide the strips into four equal blocks, with each block containing a biochar-amended micro-plot and a control (non-amended) micro-plot of 0.66 m diameter each. The biochar and control treatments were allocated randomly in the first block followed by an alternated allocation in subsequent blocks to ensure a balanced allocation and coverage of field variability in both directions of the strip. There was a buffer zone of 1.8 to 2.5 m between and around the micro-plots.

To establish the circular micro-plots, the areas were marked out with a ring. The soil was then excavated to 10 cm depth into large plastic tubs and manually mixed with biochar at 1 kg per micro-plot (equivalent to 29.2 t ha^-1^), or mixed without biochar (i.e. for the control micro-plots). Soil at the 10–12 cm depth was also excavated followed by lining of the perimeter of the micro-plots with plastic garden edging (15 cm wide; 4 mm thick) to 12 cm depth. The micro-plots were then back-filled, firstly with the soil excavated from the 10–12 cm depth and then with the biochar-soil mixture (biochar micro-plots) or the mixed soil (control micro-plots). The garden edging was kept > 2−3 cm above the soil surface to prevent lateral loss of biochar. During backfilling, the soil with or without biochar was compacted gently with a wooden driver in smaller layers (2 to 3 cm) to achieve close to original bulk density of the repacked soil (Fig B in S1 Supporting Information).

### Biochar production

Biochar was produced at 450°C by slow pyrolysis (e.g. 5–10°C min^-1^ from 20 to 450°C, 40 min residence time) from a δ^13^C-depleted *Eucalyptus saligna* biomass, comprising stem wood, branches/twigs, and leaves. The biomass was sourced from trees that had been grown under an elevated-CO_2_ environment for two years [52]. The biomass was air-dried after harvest and kept in a storage room for ~3.5 years before pyrolysis. An externally heated closed batch reactor (~10 kg batch of dry biomass), where the O_2_ ingress is restricted and any initial O_2_ disappears quickly as syn gases are evolved during heating, was used to produce biochar with liquid petroleum gas as an energy source (Pacific Pyrolysis Pty Ltd, Somersby, Australia). N_2_ gas replaced the atmosphere in the reactor during the cooling off period.

### Biochar carbon recovery in soil layers

Soils were sampled on day zero at 0−10 cm depth from the biochar-amended and control micro-plots. To track downward migration of biochar in the soil profile, soil sampling was done at different times (~4, 8 and 12 months) below the zone of application to 30 cm depth (for all sites at all times) or to 50 cm depth (for Arenosol and Cambisol at 12 months only). The soil cores were collected using a hammer-driven steel corer (cutting head diameter 32.0 or 44.3 mm) and were separated into 0–8, 8–12, 12–20, 20–30 cm, or 30–50 cm depths. The soil cores were collected from four random locations (~ 4 to 5 cm away from the collar area and garden edging) to make one composite sample per micro-plot. The holes made after coring were refilled after lining with a PVC tube. Soil bulk density was measured based on mass of dry soil collected per unit volume of core from respective soil depths. Some soil compaction was observed during soil coring at the Elliott site. Hence the soil cores were carefully sampled sequentially down the soil profile (0–8, 8–12, 12–20 and 20–30) and any soil moving from the top layer was removed from deeper soil layers to prevent biochar contamination.

A two-source C isotope mixing model was used to determine the proportion of biochar-C in total soil C [C_Biochar_ (%)]; see S1 Supporting Information.

The biochar-C stock (t ha^-1^) in each soil layer at 4, 8 and 12 months was calculated as below:

$$Biochar\;C_{Stock}=\;C_{Biochar}\left(\%\right)\;\times \;z\;\times \rho \times \;C_{total}\times \;10$$ (1)

where z (m) is the thickness of the sampled soil layer, ρ (t m^-3^) is the bulk density, and C_total_ (g kg^-1^) is the total C content of biochar-soil mixture.

Biochar-C recovery in each soil layer at 4, 8 and 12 months were calculated as below:

$$Biochar\;C_{re\text{cov}ery}(\%)=\frac{Biochar\;C_{stock}insoil}{Initialbiochar\;C}\times 100$$ (2)

where original biochar-C on day zero was applied at 19.5 t ha^-1^ to 10-cm depth.

### Biochar and soil analyses

Representative, air-dried biochar and soil samples were ground to <53–80 μm and further dried at 70°C for >16–24 h before the analyses of (i) total C and nitrogen (N) using a LECO TruMac CN analyser and (ii) δ^13^C using a Delta V Thermo Finnigan IRMS (analytical precision <0.1‰). Solid-state ^13^C cross polarization (CP) nuclear magnetic resonance (NMR) spectra of the biochar was acquired as described in Baldock et al [53]. Clay mineral composition in soils was determined by a PANalytical X'Pert Pro Multi-purpose Diffractometer. Further analyses of biochar properties (pH, EC, CEC, inorganic C, degree of aromatic condensation, organic functional groups) and soil properties (pH, EC, CEC, particles size, cations, clay mineraology) and associated analytical details are given in the SI. Description of the initial properties of biochar and soils is given in Table 1 and Table A in S1 Supporting Information.

**Table 1:** Key properties of biochar, plant and soils.

	Biochar	Arenosol	Cambisol	Ferralsol
Carbon (%)	66.79±0.22	0.66±0.04	1.67±0.20	6.25±0.17
Inorganic C (%)	0.13±0.01	−	−	−
Organic C (%)	66.66±0.25	−	−	−
H/C_org_	0.63±0.08	−	−	−
-Δδ	0.30	−	−	−
Nitrogen (%)	1.04±0.02	0.06±0.00	0.15±0.01	0.53±0.01
δ^13^C (‰)	-36.7±0.2	-24.9±0.1	-24.9±0.1	-27.0±0.1
δ^13^C of light fraction (‰)	−	-26.2±0.2	-26.2±0.1	-27.9±0.1
δ^13^C of aboveground biomass (‰)	−	-27.5±0.6	-27.5±0.6	-28.6±0.5
δ^13^C of root (‰)	−	-27.3±0.8	-27.3±0.8	-29.0±0.6
pH (1:5 H_2_O)	9.8±0.1	6.1±0.3	6.9±0.1	6.0±0.1
EC (1:5 H_2_O) (ms m^-1^)	160.0±5.1	36.2±11.8	52.3±14.3	120.0±12.2
CEC (cmol kg^-1^)	12.2±2.4	5.4±0.3	11.1±0.5	21.4±2.1
Clay (%)	−	7.6±0.3	17.8±2.3	15.1±1.7
Silt (%)	−	4.9±0.1	18.8±5.3	25.9±2.0
Fine Sand (%)	−	24.2±2.9	28.5±10.8	42.2±4.4
Coarse Sand (%)	−	61.7±3.3	32.8±15.1	13.0±0.5
Texture		Loamy fine sand	Coarse sandy loam	Fine sandy loam
Clay minerals		Kao***Sm**Go*Hem*Ant*	Kao***Sm**Go*Hem* Ill*Ant*	Kao***Go**Hem**Gib*Ant* Vem*
Ca (g kg^-1^)	−	0.8±0.1	2.4±0.6	2.4±0.3
Mg (g kg^-1^)	−	0.3±0.0	0.8±0.0	2.5±0.1
Al (g kg^-1^)	−	11.5±0.9	24.4±0.9	102.3±1.5
Fe (g kg^-1^)	−	7.0±0.5	18.2±0.5	107.5±1.3
Mn (g kg^-1^)	−	1.3±0.1	1.5±0.1	5.9±0.3

The numbers after “±” are the standard deviations (n = 3). The symbol “−” means the property was not applicable or measured. “-Δδ” means the difference between the ^13^C chemical shift of ^13^C-benzene sorbed to biochar and neat ^13^C-benzene (Δδ = δ_sorbed benzene_ − δ_neat benzene_) as an indicator of degree of aromatic condensation of biochar [21]. Inorganic C was determined by a titrimetric method [78]. “H/C_org_” is the molar ratio of hydrogen and organic C. Kao = kaolinite; Sm = Smectite; Go = goethite; Hem = hematite; Gib = gibbsite; Ill = Illite; Ant = anatase; Vem = vermiculite. The symbols “***”, “**”, “*” represent more than 60%, 5−20%, and less than 5%, respectively.

### Gas sampling for measuring total soil and biochar carbon emissions

The static alkali absorption method [54] was used to determine (i) total C emission and (ii) associated δ^13^C. In this method, the CO_2_ evolved from soil is absorbed in alkali (NaOH) placed within a static closed chamber over a specific time period (~24 h in the present study). The closed chamber was made from an opaque PVC tube (24-cm diameter and 20-cm height) and a smoked plexiglass lid was sealed to the tube. This chamber was placed on a collar (24-cm diameter and 15-cm height), which was inserted in the centre of each micro-plot to 12 cm depth. The chamber was clamped to the collar. A rubber O-ring was placed between the chamber and the collar to ensure proper sealing. The collars had holes (3 mm diameter) at the bottom 6-cm perimeter to allow root proliferation and equilibration of internal and external soil environment. The closed chambers were covered with Mylar sheeting, except in the first 2 or 3 sampling campaigns, to minimise temperature fluctuations, as confirmed by the air temperature data inside and outside the chamber. This Mylar covering also ensured a darker environment inside the chamber to minimise uptake of the respired CO_2_ by the pasture shoots emerged within 2 weeks after sowing.

A 100 mL jar (6.5 cm diameter) containing 40 mL of 2 M NaOH was placed inside the static closed chamber on a tripod stand to trap CO_2_ emission from soil. The gas samplings were undertaken at < 1 to 3 weeks interval for 31 times at the Cobbitty site and 34 times at the Elliott site over 12 months. To account for background CO_2_/^13^CO_2_ in the static headspace with an internal volume of ~ 10 L, three 10 L plastic buckets with enclosed base (i.e. no soil exposed) were installed around the micro-plots at each of the sites.

To quantify total soil CO_2_-C emission rate (TCE rate), CO_2_ in the alkali trap was determined by titrating a 1 ml aliquot of the alkali trap solution against 0.1 M HCl, using phenolphthalein as the indicator. A 10 mL aliquot of the CO_2_ trapping solution (2 M NaOH) was mixed with 10 mL of 1.0 M SrCl_2_ to form SrCO_3_ for δ^13^C analysis [21]. The TCE rate (g CO_2_-C m^-2^ d^-1^) was calculated as:

$$TCErate=\frac{mC}{A\Delta t}$$ (3)

where, mC represents the amount of CO_2_-C absorbed in the alkali trap within the time period Δt (*e*.*g*. 24 h) after the chamber was closed. *A* is the surface area of collar (0.045 m^2^).

A two-pool isotope mixing model was used to determine (i) the proportion of biochar-derived CO_2_-C in total CO_2_-C emission [biochar CO_2_ (%)] and then (ii) the proportion of added biochar-C mineralised [21]. The end members in the two-source C isotope mixing model are: (i) δ^13^C of the fresh biochar (δ_B_^13^C) and (ii) δ^13^C of soil + plant respired CO_2_ (δ_C_^13^CO_2_) from the control micro-plot.

BiocharCO2(%)=δTC13O2−δCC13O2δBC13−δCC13O2×100 (4)

where δ_T_^13^CO_2_ is the δ^13^C value of the CO_2_-C released from biochar-amended micro-plots.

The δ^13^C signatures of two C sources in the soil + plant CO_2_-C end member were relatively similar, particularly the δ^13^C signature of light C fraction and plant C (Table 1). Furthermore, the influence of biochar-plant interactions on the δ^13^C signature of the soil + plant CO_2_-C end member relative to the control soil was expected to be minimal. This is because there was a lack of significant influence of biochar on pasture growth rate except on two occasions in the Ferralsol (Fig D in S1 Supporting Information). This provided confidence in using δ^13^C signature of CO_2_-C emitted from the control micro-plots as one of the end members in Eq 4.

Biochar-C mineralisation rate was determined by multiplying the proportion of biochar-derived C [biochar CO_2_ (%)] with the TCE rate. Since there was little or no relation of the total or biochar-C emission rate with temperature (Figs E and F in S1 Supporting Information), we used a linear interpolation of emissions between the sampling points. The cumulative C emissions from total, biochar or native C sources were then calculated by summing the linearly interpolated emissions at different time points over 12 months.

### Mean residence time of biochar carbon in soil

To estimate MRT of biochar in soil, the cumulative pattern of biochar-C mineralised over 12 months was fitted to three commonly used models i.e. one-pool and two-pool exponential models and an infinite-pool power model [23, 41]; see S1 Supporting Information. These models consider an organic material comprising a single fraction (one-pool), a labile and a recalcitrant fraction (two-pool), or a continuum of fractions (infinite-pool) with exponentially decreasing decay rates. Thus, fitting these contrasting models would indicate whether including additional pools in the calculation may improve model fitting and hence the estimates of biochar MRT in the soils.

### Statistical analysis

For determining the relationship of total C and biochar-C emission rates with temperature and/or moisture, a log10 transformation was applied to the C emission data. Average soil temperature data over the 24 h period were used in the relationships. First order differences were computed to remove non-stationarity in the measured variables, that is, the relationship between the change in log10 (Δ*log*_10_) of C emission rates (*R*) and the change in temperature (Δ*T*) and/or moisture (Δ*M*) was examined. A mixed model was fitted to Δ*log*_10_ *R* through the origin (zero intercept) comprising linear effects of Δ*T* and/or Δ*M*, with exponentially correlated errors relative to the time interval between measurement points.

A mixed model analysis of soil C content, C/N ratio, δ^13^C and bulk density for each soil and time was undertaken. Each mixed model consisted of fixed treatment, depth and treatment by depth effects, random replicate effects and auto-regressively correlated errors for depth (within plot) [55]. Total biochar-C recovery across 0−12, 0−30 and/or 0−50 cm depths was analysed using analysis of variance (ANOVA) with fixed effects of replicate and time for each soil separately. Biochar-C recovery below 12 cm soil depth was analysed using a mixed model with fixed effects of soil type, time and their interaction, and random effects of replicate (within soil), and allowing separate residual variances for each soil. When the F-statistic was significant, the means were compared using an *LSD* test at 5% significance unless stated otherwise.

## Results

### Total carbon emission

**Fig 1:**
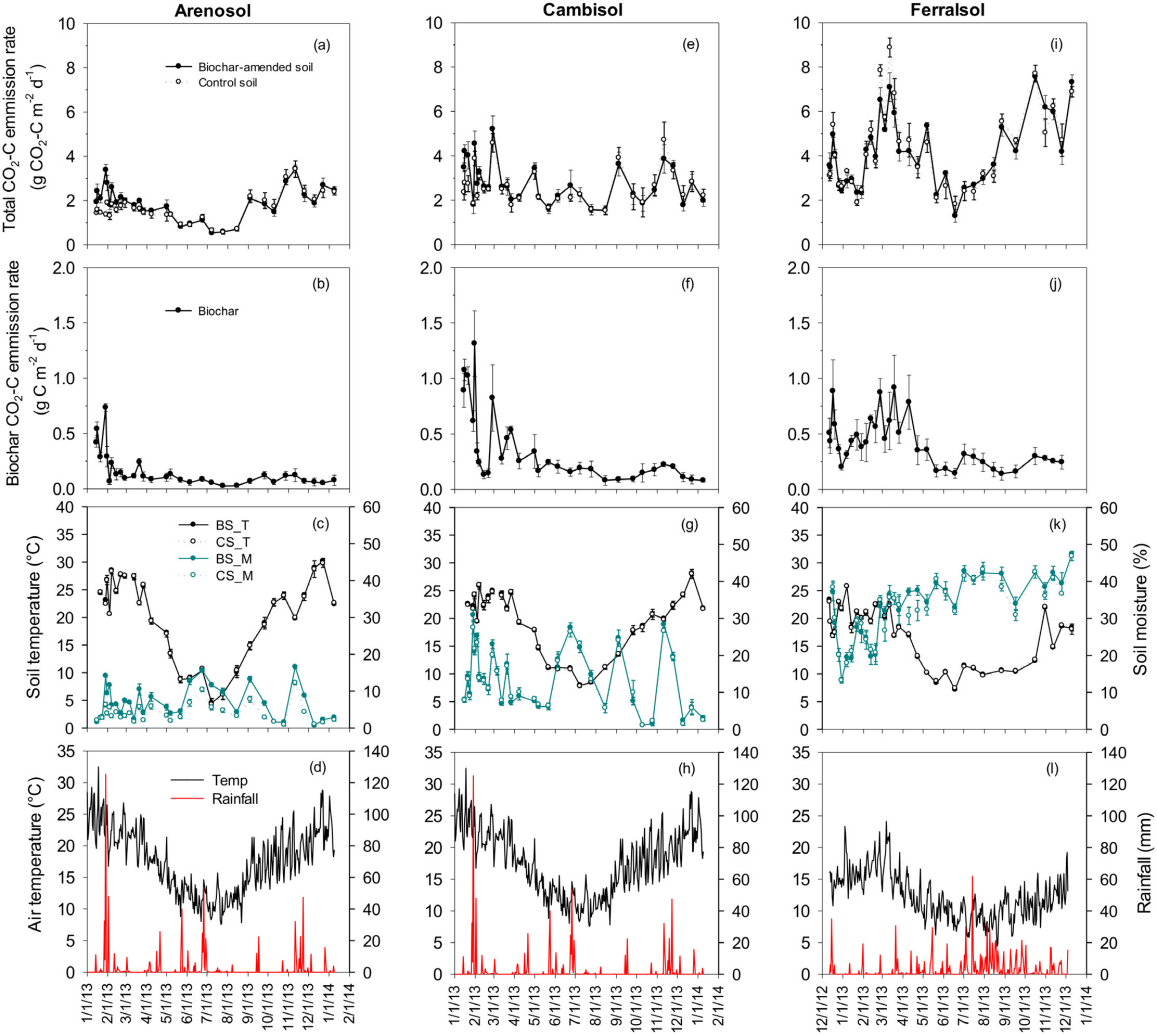
Total daily CO_2_-C emission and biochar-C mineralisation rates in the biochar-amended and control Arenosol, Cambisol and Ferralsol. The bottom two panels includes soil temperature at 5-cm depth, volumetric soil water content at 0−10-cm depth, mean air temperature (an average of maximum and minimum) and rainfall over the 12 months at the Cobbitty field site in New South Wales and at the Elliot site in Tasmania. The weather data were downloaded from the nearest weather station at the Camden airport (http://www.bom.gov.au/) and University of Tasmania (http://console.weatherdata.com.au/index.html). The symbols of biochar-amended and non-amended (control) micro-plots are filled and empty circles, respectively. Error bars are ± standard errors (n = 4).

The total C emission rates were similar in the biochar-amended and control micro-plots throughout, except in the Arenosol where greater C emission rates were observed in the biochar amended *vs*. control micro-plots during the first 1.5-months (Fig 1). Total C emission was in the order of Arenosol < Cambisol < Ferralsol. Correlations between total C emission rates and temperature or moisture individually were low. However, when temperature and moisture were included together in the model, the R^2^ values were increased, more for the Arenosol than Cambisol but not for the Ferralsol (Fig E in S1 Supporting Information). This can be explained by the negative correlation between temperature and moisture in the Arenosol and Cambisol (Table B in S1 Supporting Information). The greater increase of total C emission rate in the Ferralsol relative to other two soils from August to November seemed to be related to a rapid increase of plant growth rate during this period (*cf*. Fig 1 and Fig D in S1 Supporting Information).

### Biochar carbon mineralisation in soil

The biochar-C mineralisation rates varied between 0.03 and 1.30 g CO_2_-C m^-2^ d^-1^ over 12 months and were higher in the first 3 to 4 months, albeit fluctuated greatly, than in the later period across all sites (Fig 1). There was little or no correlation between biochar-C mineralisation rates and temperature and/or moisture (Fig F in S1 Supporting Information). Over 12 months, 2.0% of the added biochar-C was mineralised in the Arenosol, 4.6% in the Cambisol and 7.0% in the Ferralsol (Table 2).

**Table 2:** The proportion of biochar-C mineralised over 12 months and the mean residence time (MRT) of biochar-C in Arenosol, Cambisol and Ferralsol.

Soil type	Duration (day)	Biochar-C mineralised (%)	One-pool		Two-pool		Infinite-pool	
			MRT (yr)	R^2^	MRT (yr)	R^2^	MRT (yr)	R^2^
Arenosol	360	1.96±0.13	44	0.817	71	0.997	1079	0.994
Cambisol	360	4.56±0.55	18	0.830	39	0.997	172	0.993
Ferralsol	349	7.03±0.39	11	0.942	29	0.990	21	0.980

The numbers after the ± are standard errors (n = 4)

### Carbon emission from native soil and plant sources

Cumulative C emission over 12 months from the combined native soil and plant sources from the biochar-amended micro-plots was similar (Arenosol and Cambisol) or lower (Ferralsol; *p* < 0.01) compared to the control micro-plots (Fig G in S1 Supporting Information). Like total C emission rates from the biochar-amended micro-plots, correlations between native soil C emission rates and temperature or moisture individually were low; however the R^2^ values were increased (Arenosol > Cambisol) in a combined model of temperature and moisture (Fig E in S1 Supporting Information).

### Biochar influence on soil carbon

**Fig 2:**
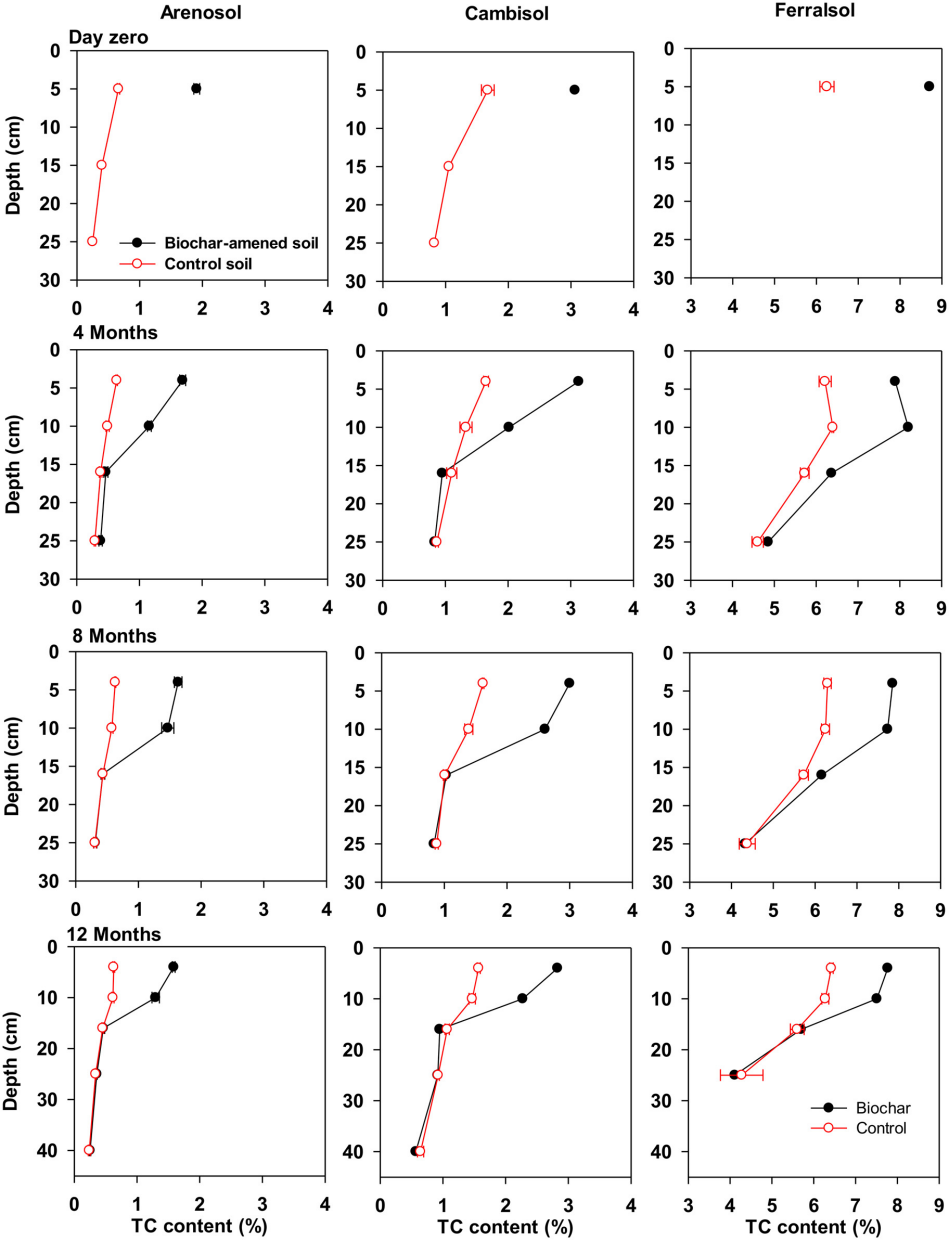
Total carbon (TC, %) content of biochar-amended and control Arenosol, Cambisol and Ferralsol. The symbols of biochar-amended and non-amended (control) micro-plots are black circle and red empty circle, respectively. The data are presented at different depths and times after biochar incorporation in the soils. Error bars are ± standard errors (n = 4).

The application of biochar (day zero) significantly (*p* ≤ 0.008) increased soil C content in the 0–10 cm depth, i.e. from 0.7% to 1.9% in the Arenosol, 1.7% to 3.2% in the Cambisol, and 6.3% to 8.6% in the Ferralsol (Fig 2). Correspondingly, the calculated soil C stocks increased from 10.3 to 29.6 t ha^-1^ in the Arenosol, 20.9 to 40.1 t ha^-1^ in the Cambisol, and 50.0 to 69.2 t ha^-1^ in the Ferralsol in the applied 0−10 cm soil layer. At 4, 8 and 12 months, total soil C contents were significantly (*p* ≤ 0.006) greater in the biochar-amended soils than the corresponding controls at the 0−8 cm and 8−12 cm soil layers. Whereas, no significant difference was found in the subsoil layers (12−20, 20−30 cm) between the biochar-amended soils than the corresponding controls, except in the Ferralsol where biochar micro-plots had higher total soil C in the 12−20 cm layer at 4 and 8 months than the control (Fig 2).

### Biochar carbon recovery and downward migration

**Fig 3:**
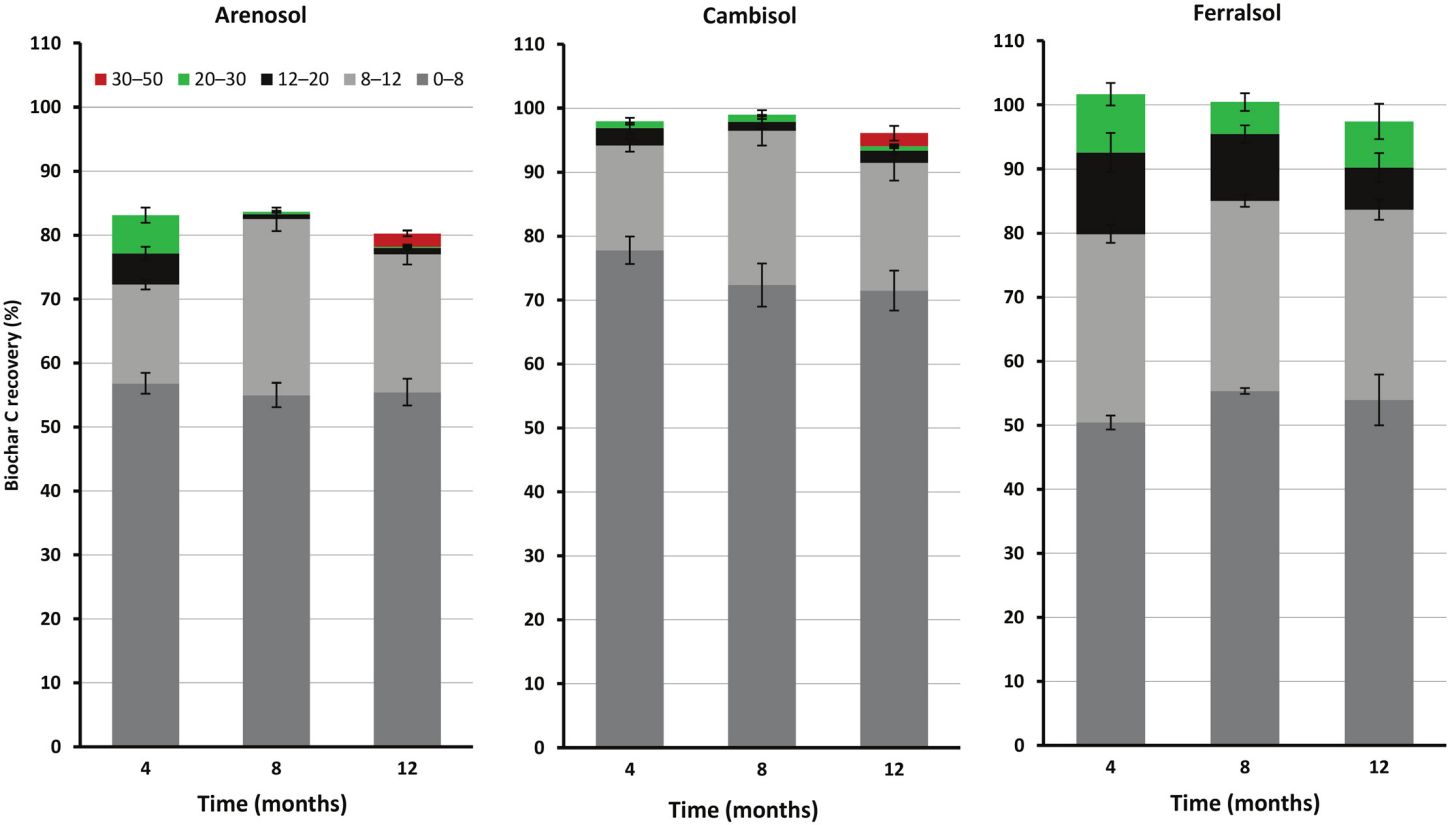
Biochar-C recovery (%) at different depths and times (4, 8 and 12 months) in the biochar-amended Arenosol, Cambisol and Ferralsol of the surface (0–10 cm) applied biochar-C on day zero. The data are presented at different depths and times after biochar incorporation in the soils. Error bars are ± standard errors (n = 4).

At day zero, the recovery of biochar-C in the applied 0−10 cm depth was 95.8%±2.0%, 100.6%±2.4% and 104.1%±1.8% in the biochar-amended Arenosol, Cambisol and Ferralsol, respectively. The total recovery of biochar-C across 0–30 or 0–50 cm depths was significantly (*p* < 0.05) decreased with time (at 4, 8 or 12 months compared to day zero) in the Arenosol only. When comparing biochar recovery at 0–12 cm depth during 4 to 12 months (relative to day zero), both Arenosol (72.3–82.5%) and Ferralsol (79.8–85.1%) had a significant decrease, but not the Cambisol (91.4–96.4%). The proportion of biochar-C recovered in the deeper soil depths (below 12 cm) varied with soil and time (p < 0.05), *i*.*e*. decreased in the Arenosol and Ferralsol but not in the Cambisol (Fig 3). At 4 months, the recovered proportions from the 12−30 cm depth (combined across 12−20 and 20−30 cm) varied among the soils as: 10.9% (Arenosol), 3.8% (Cambisol) and 21.8% (Ferralsol) (Fig 3). After 8−12 months, the recovered proportion in the 12−30 cm depth decreased to 1.2%, 2.5–2.7% and 13.8–15.4% in the biochar-amended Arenosol, Cambisol and Ferralsol, respectively. Furthermore, 2.1% and 2.0% of the applied biochar-C was recovered in the 30−50 cm depth after 12 months in the Arenosol and Cambisol, respectively. At 12 months, including all sampling depths, between 80.3%±2.7%, 96.1%±4.3% and 97.4%±4.6% of the initial biochar-C was recovered in the Arenosol, Cambisol and Ferralsol, respectively (Fig 3). When including total biochar-C mineralised (Table 2) and the recovery of biochar in the soil profile to 30 cm in the Ferralsol or 50 cm in the Arenosol and Cambisol (Fig 3), the total recovery were 82.2%±2.3%, 100.7%±2.9% and 104.4%±4.9% in the Arenosol, Cambisol and Ferralsol, respectively. The amounts of biochar-C in the deeper soil layers were 1.7 and 2.2 times higher than the total mineralised biochar-C in the Arenosol and Ferralsol, respectively (Table 3).

**Table 3:** *In-situ* fate of applied biochar in the Arenosol, Cambisol and Ferralsol after 12 months. Losses of biochar to deeper layers (below 30 or 50 cm depth) were not measured.

	Arenosol	Cambisol	Ferralsol
*The fate of applied biochar-C in soil*			
Mineralised as CO_2_-C (t ha^-1^)	0.38±0.02	0.89±0.11	1.37±0.08
Move to soil layers (t C ha^-1^)			
12–20 cm	0.19±0.04	0.38±0.12	2.03±0.27
20–30 cm	0.04±0.01	0.13±0.06	0.98±0.27
30–50 cm	0.41±0.09	0.40±0.22	na
*Native plant and soil C emissions*			
Biochar-amended soil (t ha^-1^)	5.85±0.20	8.27±0.79	13.30±0.29
Control soil (t ha^-1^)	5.96±0.36	9.11±0.72	14.80±0.66

‘na’ is not analysed. The numbers after “±” are standard errors (n = 4).

## Discussion

### The persistence of biochar carbon in soils under field conditions

In this field-based study, the observed mineralisation proportions of the applied biochar-C (between 2.0 and 7.0% over 12 months) were higher than those previously reported (i.e. 0.7 and 2.7% over 12 months) in plant free laboratory studies using comparable *E*. *saligna* biochars across contrasting soil types [21, 22]. These results suggest that the presence of plants and associated supply of labile C inputs *via* roots could increase biochar-C mineralisation through increased microbial activity and enzyme production [24, 34, 56–58]. Another reason for higher mineralisation under field compared to laboratory conditions is the higher fluctuations in soil temperature (*e*.*g*.*≥* 19–29°C at each site) and moisture (≥16–35% in all soils). These environmental fluctuations could increase physical weathering and chemical or biological degradation of biochar [59, 60]. In addition to the influence of root C input and environmental fluctuations, the NMR characterisation of the wood biochar used in this field study shows a higher proportion (33% *vs*. ~16%) of nonaromatic C and a lower degree of aromatic condensation (0.3 *vs*. ~0.8) compared to the wood biochar used by Singh et al. [21].

**Fig 4:**
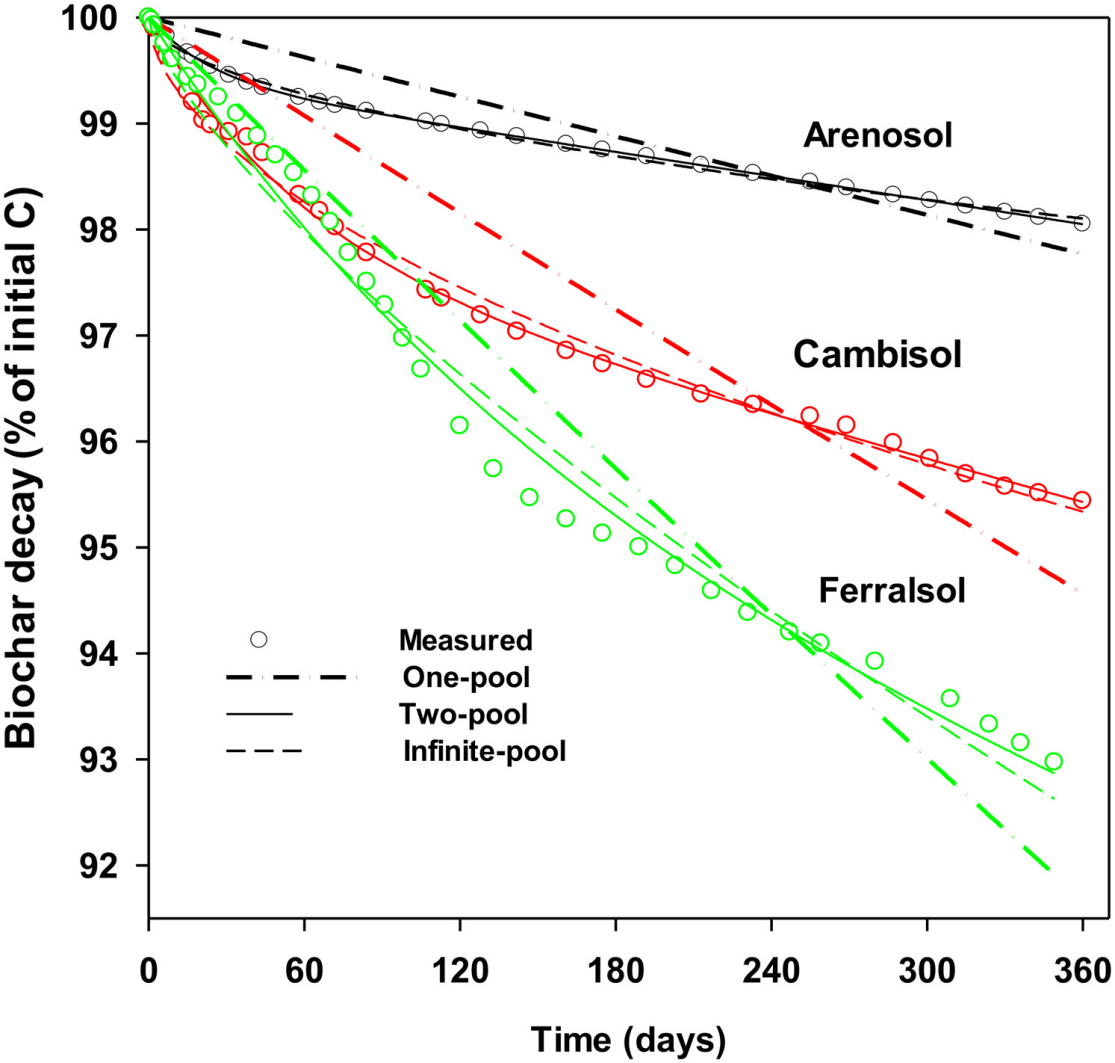
Percent of biochar-C decayed in Arenosol (black), Cambisol (red) and Ferralsol (green) over 12 months. The three models employed to estimate mean residence time of biochar-C in soil were (i) one-pool exponential (dotted line), (ii) two-pool exponential (solid line) and (iii) infinite-pool power model (dash line).

The calculated MRT varied, depending on the model type, from 11−44 years (one-pool exponential), 29−71 years (two-pool exponential) or 21−1079 years (infinite-pool power model) (Table 2). The model fit was strongest for the two-pool model and weakest for the one-pool model (Fig 4), supporting previous conclusions that biochar is not a single pool of C but is better described as a labile C pool which mineralises rapidly, and a recalcitrant C pool with a much slower rate of mineralisation [41]. Data from this study demonstrated that 54–62% of the total biochar-C mineralisation occurred with the first 4 months across all three sites (Fig H in S1 Supporting Information). It is possible that biochar-C mineralisation over 12 months may still be originating from more labile (e.g. alkyl) compared to recalcitrant (e.g. aryl) functional groups within the biochar. Hence, longer term C mineralisation observations are warranted to obtain robust estimates of biochar MRT. The infinite-pool model considers biochar as a continuum of substrates of increasing chemical recalcitrance and decreasing decay rates [61]. Thus, this model may be more appropriate where longer term measurements are available to parameterise the model and obtain a more precise estimates of biochar MRT [41]. There is however a need for assessing sources of bias and uncertainty associated with the prediction of biochar MRT relative to the duration of biochar-C mineralisation data across different model types [41].

Compared to the relatively long MRT (several 100 years) estimated by Fang et al. [22], this field-based study estimated far shorter MRT of biochar-C (i.e. a few decades) using the two-pool exponential model. These results demonstrate that field MRT estimates cannot be simply extrapolated from laboratory studies, as proposed by Kuzyakov et al. [24, 62] who suggested that a 10 times increase in MRT of biochar-C might be expected in field conditions of lower temperature and microbial activity compared to controlled laboratory studies. Studying the same biochar as used in this study, Weng et al. [38] reported MRT estimates of 351–449 years (using the two-pool model) in a sub-tropical, clay rich (44%), acidic (pH 4.5) Ferralsol. The MRT estimates in the subtropical system are 12–15 times higher than in the temperate Ferrasol with more moderate pH (6.0) and clay content (15%) used in our study (Table 2). Thus, instead of a more rapid mineralisation (decreasing persistence) of biochar-C in a warmer environment, it was slower. The specific properties of the subtropical Ferralsol in Weng et al. [38] which may have led to slower biochar-C mineralisation are not known, but may relate to greater protection through biochar-clay interaction and incorporation into soil aggregates [22, 39, 40].

### Impact of environment and soil type on biochar carbon mineralisation

Our results show little correlation between biochar-C mineralisation and environmental factors, such as temperature and moisture (see S1 Supporting Information). Moreover, biochar-C mineralisation was greater in the colder Elliott (TAS) site compared to the warmer Cobbitty (NSW) site. These results suggest other site-specific factors (e.g. soil properties and plant growth rate) play a more important role in impacting biochar-C mineralisation relative to the environment.

Within the one field environment (Cobbitty site, NSW), we observed 57% lower biochar-C mineralisation in the low C and low clay Aerenosol compared to the relatively C- and clay-rich Cambisol. In this case the results suggest that higher native soil C and plant growth and associated labile-C inputs in the Cambisol would outweigh any protective effects on biochar-C mineralisation that may arise from biochar-clay interactions. Furthermore, across the three soils, we observed a positive correlation between biochar-C mineralisation and total soil C content (Fig I in S1 Supporting Information), with the C content higher in the soils with higher clay content (Table 1). On the other hand, Bruun et al. [63] reported a positive correlation between clay content (11−23%) and biochar-C mineralisation in soils with similar native C content. However, in our study, we did not observe a decrease in biochar-C mineralisation with increasing clay content from 8% to 18%, possibly due to the stronger positive priming effect of native SOC and plant C input [34, 56] relative to the stabilisation effect of the organo-biochar-clay interaction [38].

The greatest proportion of biochar-C mineralised in the Ferralsol among the three soils in the present study contrasted with the results of Fang et al. [22, 64], who reported lower mineralisation of biochar-C in an Oxisol (Ferralsol) than other soil types (Inceptisol, Vertisol, Entisol). In our study, the clay content in the Ferralsol is only 15.1% and the dominant clay type is kaolinite, which may have limited the extent of biochar-clay interaction relative to Ferralsols containing 30−44% clay content or dominating Fe and Al oxides in other studies [22, 32, 38]. In addition to the influence of high native SOC content, the low persistence of biochar in the Ferralsol (Elliott site, TAS) could also be due to its high moisture content, pasture growth rate or fertiliser N input. These site-specific factors may increase microbial biomass (Fig C in S1 Supporting Information) and earthworm abundance/activity (see SI) and consequently resulting in greater biochar-C mineralisation in the Ferralsol, relative to the Cambisol or Arenosol [65].

### Downward migration of biochar in soil profile

In the field situation, biochar could be prone to losses from the applied layer via lateral and/or verticle movements. In this study, we minimised the opportunity for lateral surface loss of biochar from the repacked soil by enclosing the microplot area with a ~3 cm raised edge. The results show that only ~82% of the applied biochar-C was recovered in the Arenosol, while 101% and 104% were recovered in the Cambisol and Ferralsol, respectively, after accounting for its loss *via* mineralisation and recovery in the soil profile (up to 50 cm). These results suggest that biochar is likely to have migrated in the Arenosol through consistent vertical movement to below 50 cm [27, 47]. The downward migration of biochar in the Arenosol could have been due to movement of finer biochar particles or labile dissolved components, which would be facilitated by rainfall events, such as the heavy rain event (125 mm d^-1^) two weeks after biochar incorporation in the Cobbitty site. The lower concentration of clay in the Arenosol would have resulted in lower organo-biochar-mineral complexes [47], thus minimising incorporation of biochar into stable aggregates. Our results of high downward migration of biochar in the Arenosol are consistent with those reported by Haefele et al. [44] who found that nearly half of the applied rice-husk biochar-C moved to below 30 cm depth over a four-year study in a sandy soil.

The full recovery of the applied biochar-C in the Cambisol and Ferralsol to 30 or 50-cm depth (Table 3; Fig 3) is consistent with the study by Singh et al. [28] reporting > 99% recovery of the surface (0−2 cm) applied biochar in a clay loam soil (Cambisol) to 15-cm depth. The Cambisol and Ferralsol might have a high abundance of meso- and micro-pores, which would favour retention of biochar particles [47]. Furthermore, biochar particles and associated dissolved organic matter could interact with the functional groups of clay minerals in these soils to create less mobile organo-mineral complexes [39, 66]. For example, reactive Fe and Al oxides, present in Ferralsols, are regarded to have stronger interaction (e.g. *via* ligand exchange) with biochar and organic matter compared to other clay minerals such as kaolinite and smectite [64, 67]. However, the Ferralsol mineraology did not prevent migration, with 13.7% of the applied biochar-C recovered below 12 cm depth, compared to 4.6% in the Cambisol (Fig 3). This may relate to greater bioturbation by earthworms [68] and higher annual precipitation rate (1109 *vs*. 788 mm) in the Ferralsol (Elliott site) *vs*. Cambisol (Cobbitty site). The greater earthworm abundance in the biochar-amended *vs*. the non-amended Ferralsol, or in the other two soils (Cambisol and Arenosol), suggests that wood biochar produced by slow pyrolysis may favour earthworm abundance and activity in a soil system where they exist. Indeed, Van Zwieten et al. [69] showed significantly increased abundance of earthworms in a biochar-amended Ferralsol *vs*. control using an earthworm avoidance assay. These earthworms can ingest finer biochar particles and consequently facilitate their physical mixing and redistribution vertically and/or laterally into the soil profile *via* bioturbation [65].

The demonstrated downward migration of biochar needs to be considered when aiming to estimate biochar MRT. The vertical distribution of biochar in the soil may influence its persistence since deeper soil layers generally have lower microbial activity and may have higher clay content [70] relative to the top soil layer. Indeed, Fierer et al. [71] found that microbial biomass C in deep soil (i.e. 15−25 cm) was 2−9 times lower than in top soil (0−5 cm) and the proportions of fungi, the major decomposers of aromatic compounds [72], were highest at the soil surface and substantially lower in the subsurface. Furthermore, decreased plant C input in deeper soil layers (e.g. root density decreases with increasing soil depth) [73] could increase the persistence of biochar in soil.

### Biochar effect on C emission from native plant and soil sources

The use of a two-source isotopic mixing model to distinguish C sources in a biochar-treated soil is challenging at the natural abundance level, particularly where plant contributions could serve as a third C source [74]. Nevertheless, C-isotopic approaches have been successfully employed to determine contributions of different C sources to total CO_2_ emission. This is particularly possible in studies where (i) biochar-plant root interactions are non-significant, or deemed to have a non-significant impact on the δ^13^C signature of soil CO_2_ efflux [57]; and/or (ii) the δ^13^C signatures of plant-derived and soil C sources are similar, e.g. both sources comprise either C_4_-dominated [14, 34] or C_3_-dominated δ^13^C signatures (this study). Although the two-source C isotopic model [27, 57, 74] limits our ability to separately quantify C emission from plant or soil sources, this approach still enhanced the understanding of the interactions of biochar with the plant-soil systems under the field conditions.

Since the pasture growth rate was higher (up to 60%) in the biochar-amended versus the non-amended Ferralsol on two occasions (Fig D in S1 Supporting Information), thus belowground C input, plant respiration and any root C input associated positive priming of native soil C [27, 75, 76] are likely to be greater. However, our results of consistently (*p* < 0.01) decreased C emissions from the plant-soil sources in the presence of biochar in the Ferralsol suggest the occurrence of negative priming of native soil or plant-derived C by biochar. This could be due to biochar-induced increases in interactions between native organic matter and reactive clay mineral *via* ligand exchange in the Ferralsol relative to other soils [22, 64, 77]. The greater polyvalent cations (Ca, Mg, Al, Fe, and Mn) reactivity of the Ferralsol versus the Arenosol and/or Cambisol (Table 1) could have also enhanced the development of organo-mineral interactions in the presence of biochar *via* cation bridging. Interestingly, the decreased native C emission (by 1.50 t ha^-1^) from plant-soil sources in the presence of biochar was larger than the amount (1.37 t ha^-1^) of biochar-C mineralised over 12 months (Table 3). These results would have positive implications for the overall C budget in the pasture systems under the Ferralsol. The lower native SOC mineralisation by biochar cannot be attributed to a lower microbial biomass which was 37% higher in the biochar-amended *vs*. control Ferralsol (Table C in S1 Supporting Information). There were occasions of consistently higher native C emissions from the biochar-amended *vs*. the non-amended Arenosol and this could be related to the biochar-induced increase in volumetric water content in the Arenosol, except during extreme dry conditions (Fig 1).

## Conclusions and Implications

The novel use of ^13^C-labelled biochar (depleted in δ^13^C relative to soil) in field settings has provided insights into the persistence and fate of biochar-C, or PyC particularly if biochar is considered as a surrogate material, across contrasting soils and environments. Between 2.0 (Arenosol), 4.6 (Cambisol) and 7.0% (Ferralsol) of the biochar-C were mineralised in 12 months. The MRT of biochar, based on the short-term study that principally accounts for mineralisation of relatively labile biochar-C components, has been estimated to vary between 20 and >1000 years, depending soil and model type. The MRT of biochar-C was the longest in the Arenosol and shortest in the Ferralsol. The higher native soil C content, earthworm abundance, N fertilisation and/or pasture growth rate may have contributed to greater biochar-C mineralisation in the Ferralsol (relative to the Arenosol or Cambisol). Our results also suggest that the positive priming effect of high SOC content on biochar-C mineralisation could be offset by the biochar-induced stabilisation of SOC in the Ferralsol. Our findings demonstrate that biochar can migrate vertically into the soil profile (≥ 30–50 cm depth over 12 months) in the order of Arenosol (NSW) > Ferralsol (TAS) > Cambisol (NSW). This is possibly due to direct leaching and infiltration of biochar in soluble or particulate forms, which can be significant in high rainfall areas, in a coarse textured soil (e.g. Arenosol), and/or *via* ingestion and bioturbation by earthworms. Our study suggests that a careful consideration of site specific characteristics, and natural physical (weathering), chemical (oxidation) and biological (bioturbation) processes, is necessary to optimise biochar production process and application strategy to maximise long-term C sequestration within soil systems. These processes may result in considerable loss of biochar-C via enhanced mineralisation or migration to deeper soil layers or waterways, particularly in a sandy soil such as the Arenosol. It may be prudent to produce biochars for soil application that have high physical strength and hence are resistant to possible slaking, oxidation or biological degradation impacts under natural conditions. The knowledge acquired from this research on the mineralisation, persistence and downward migration of biochar in contrasting soils has relevance for C models to assess its C sequestration potential in managed temperate pasture systems with implications for the global C budget.

## Supporting Information

S1 Supporting Information (DOCX)
