# Supplementary material for: In Situ Persistence and Migration of Biochar Carbon and Its Impact on Native Carbon Emission in Contrasting Soils under Managed Temperate Pastures
Source: PLoS One. 2015 Oct 28;10(10):e0141560. doi: 10.1371/journal.pone.0141560 (PMC4624795; doi:10.1371/journal.pone.0141560)
Supplement: S1 Supporting Information — (DOCX) [file pone.0141560.s001.docx]

# S1 Supporting Information

***In situ Persistence and Migration of Biochar Carbon and its Impact on Native Carbon Emission in Contrasting Soils under Managed Temperate Pastures***

**Bhupinder Pal Singh^1*^, Yunying Fang^1^, Mark Boersma^2^, Damian Collins^1^, Lukas Van Zwieten^3^, Lynne M Macdonald^4^**

**1** NSW Department of Primary Industries (DPI), Elizabeth Macarthur Agricultural Institute, Menangle, NSW 2568, Australia

**2** University of Tasmania, Tasmanian Institute of Agriculture, Burnie, Tasmania 7320, Australia

**3** NSW DPI, Wollongbar Primary Industries Institute, Wollongbar, NSW 2477, Australia

**4** CSIRO Agriculture, Glen Osmond, South Australia 5064, Australia

*Corresponding author

Email: [bp.singh@dpi.nsw.gov.au](mailto:bp.singh@dpi.nsw.gov.au)

## Materials and Methods

**Environmental factors**

During the experimental period, the field site in Cobbitty had a mean air temperature of 17.3° C yr^-1^ and a mean precipitation of 788.4 mm yr^-1^, and the field site in Elliott had a mean air temperature of 12.3° C yr^-1^, and a mean precipitation of 1109.4 mm yr^-1^.

**Biochar carbon recovery in soil layers**

A two-source C isotope mixing model was used to determine the proportion of biochar-C in total soil C [(C_Biochar_ (%)) [1]. The end members in the two-pool C isotope mixing model are: (i) δ^13^C of biochar and (ii) δ^13^C of native soil C from the control micro-plot.

$C_{Biochar}(\%)=\frac{\delta_{T}{}^{13}C-\delta_{C}{}^{13}C}{\delta_{B}{}^{13}C-\delta_{C}{}^{13}C}\times100$ (1)

where δ_T_^13^C is the δ^13^C value of total C of biochar-soil mixture, δ_C_^13^C is the δ^13^C value of native soil C without biochar amendment, and δ_B_^13^C is the δ^13^C value of fresh biochar.

### Biochar and soil analyses

The pH of biochars and soils was measured in 1:5 w/v ratio in deionised water using a pre-calibrated pH meter. Electrical conductivity (EC) was measured in 1:5 w/v extracts using a pre-calibrated EC meter. Cation exchange capacity (CEC) was determined by the compulsive exchange method [2]. Soil particle size distribution was determined by the hydrometer method. The cations (Ca, Mg, Al, Fe, Mn) in soils were determined by inductively coupled plasma-mass spectrometer with a octopole reaction system (Agilent 7500cx) following the standard aqua regia (HClO_4_/HNO_3_) digestion procedure [3]. Total C and N analysed using a Delta V Thermo Finnigan IRMS were similar to those reported by the LECO TruMac CN analyser.

X-ray Diffraction (XRD) analysis of clay minerals in soil was performed with a PANalytical X'Pert Pro Multi-purpose Diffractometer using Fe filtered Co Kα radiation, automatic divergence slit, 2° anti-scatter slit and fast X'Celerator Si strip detector. The diffraction patterns were recorded from 3 to 80° in steps of 0.017° 2 theta with a 0.5 second counting time per step for an overall counting time of approximately 35 minutes. Qualitative analysis was performed on the XRD data using in-house XPLOT and HighScore Plus (from PANalytical) search /match software.

Solid-state ^13^C cross polarization (CP) nuclear magnetic resonance (NMR) spectra of the biochar was acquired (3.2 us, 195 W, 90^o^, delay 1s) on a 200 Avance spectrometer (Bruker Corporation, Billerica, MA, USA) equipped with a 4.7 T wide bore superconducting magnet with a resonance frequency of 50.33 MHz Chemical shifts were externally referenced to the methyl resonance of hexamethylbenzene (17.36 ppm) and a Lorentziam line broadening (50 Hz) applied [4].

Earthworm abundance per square meter was determined from the number of earthworms recovered in soil cores sampled from all depths at 8 months after biochar application. It should be noted that the garden edging around the micro-plots may have restricted earthworm access/activity and hence their abundance may not be representative of an open field condition. The earthworms were found in the Elliott micro-plot site (Ferralsol), with the abundance of 698±49 m^-2^ in the biochar micro-plots and 174±116 m^-2^ in the control micro-plots to 30 cm depth. There were no earthworms in the Arenosol and Cambisol.

### Temperature sensitivity of carbon emissions

The temperature sensitivity (Q_10_) of the C emission rates, defined as the rate of increase in C emission with a 10° C increase in temperature [5], was calculated by the following approach.

For the temperature only model, the linear model was:

$\Delta log_{10}R=b\times\Delta T$, (5)

where *R* = C emission rate, *T* = temperature and *b* is the slope.

By taking exponents, we obtained the following two equations:

$10^{\Delta log_{10}R}=10^{b\times\Delta T}$and (6)

$\frac{R_{2}}{R_{1}}=(10^{b})^{\Delta T}$, (7)

where R_1_ and R_2_ are the C emission rates at two temperatures.

The Q_10_ of soil and biochar-C emission rates was calculated as:

$Q_{10}=(10^{b})^{10}$. (8)

The corresponding approximate SE using a Delta approach was calculated as:

$SE\left(Q_{10}\right)=10log_{e}10\times Q_{10}SE\left(b\right)$. (9)

### Aboveground biomass yield and analyses

Aboveground biomass samples have been collected using hand clippers/scissors twice after the establishment of pasture sward (i.e. at 4 to 6 weeks interval); the pasture plants were cut to 5 cm height. Dry weight of the biomass was measured after oven drying for 2 to 3 days at 70 °C.

### Mean residence time of biochar in soil

The Solver add-in tool in Microsoft Excel was used to estimate model parameters by minimising the sum of the squared errors between modelled and measured values [1].

The equation to estimate the proportion of biochar mineralised by a one-pool exponential model is given below [6]:

$C_{Bt}(\%)=C\times(1-e^{-kt})$ (2)

where C_Bt_ (%) the cumulative proportion of added biochar-C mineralised at time (t); k is the mineralisation rate constant. The mean residence time (MRT) is the inverse (1/k) of the mineralisation rate constant.

The equation to estimate the proportion of biochar mineralised by a two-pool exponential model is given as below [1]:

$C_{Bt}(\%)=C_{L}\times(1-e^{-k_{L}t})+(100-C_{L})\times(1-e^{-k_{R}t})$ (3)

where C_Bt_ (%) is the cumulative proportion of added biochar-C mineralised at time (t); C_L_ and (100− C_L_) are the proportions of labile (easily-mineralisable) and recalcitrant (slowly-mineralisable) pools in biochar-C, respectively; k_L_ and k_R_ are the mineralisation rate constants for the labile and recalcitrant pools, respectively. The mean residence time (MRT) is the inverse ($1/{k_{L}}$ or $1/{k_{R}}$) of the mineralisation rate constant.

The equations to estimate the proportion of biochar C mineralised by an infinite-pool power model and biochar MRT are given as below [7]:

$C_{Bt}\left(\%\right)=100-\left(100\times\frac{1-e^{-b}}{m+1}\right)\times t^{\left(m+1\right)}$ (4)

$C_{half-life}=\frac{m+1}{2\times\left(1-e^{-b}\right)}^{\frac{1}{m+1}}$ (5)

$\text{MRT}=\frac{C_{half-life}}{\ln\left(2\right)}$ (6)

where C_Bt_ (%) is the cumulative proportion of added biochar-C mineralised at time (t); m and b as the slope and intercept, respectively.

## Results

## Environmental factors

Soil temperature at the 5 cm depth varied between 4.3 and 30.2° C in the Arenosol, 4.3 and 28.1° C in the Cambisol, and 7.1 and 25.8° C in the Ferralsol. The volumetric soil water content at the 10 cm depth varied between 0.7 and 16.6% in the Arenosol, 1.1 and 30.9% in the Cambisol micro-plots and 13.2 and 47.4% in the Ferralsol during the 12 month sampling campaigns (see Fig 1 in the main paper).

## Biochar and soil properties

The NMR spectra showed that biochar contained 67.3% of total aryl C (comprising ~10.1% of O-aryl) and 29.5% of total alkyl C across alkyl, N-alkyl/methoxyl, O-alkyl, and Di-O-alkyl functional groups (S1 Table and S1 Fig). Total C content and δ^13^C signature of biochar were 66.8% and -36.7‰, respectively. Total C contents of the three soils varied between 0.7 and 6.3%, and the δ^13^C signatures varied between -24.9‰ and -27.0‰. The δ^13^C signatures of the light C fraction varied between -26.2 and -27.9‰ across the three soils, which were similar to the δ^13^C signatures (-27.3 to -29.0‰) of the aboveground and belowground parts of the pasture plants. The clay content was highest in the Cambisol (17.8%), followed by the Ferralsol (15.1%) and the lowest in the Arenosol (7.6%). The dominant clay minerals (>60%) were kaolinite in the Arenosol, Cambisol and Ferralsol; and the minor clay minerals (5−20%) were smectite in the Arenosol and Cambisol, and hematite and goethite in the Ferralsol (see Table 1 in the main paper).

### Soil bulk density at different depths, i.e. 0−8 cm, 8−12 cm, 12−20 cm, 20−30 cm or 30−50 cm, varied between 1.3−1.7, 1.4−1.7 and 0.8−0.9 g cm^-3^ in the Arenosol, Cambisol and Ferralsol, respectively (S2 Fig). Biochar significantly decreased soil bulk density in the Cambisol in the 0−8 cm and 8−12 cm depths at 8 months and 0−8 cm at 12 months (S2 Fig). The addition of biochar significantly (*p* ≤ 0.001) decreased soil δ^13^C values in the 0−8 cm and 8−12 cm soil layers (S3 Fig). In the deep soil layers (12−20 cm and 20−30 cm), a slightly more depleted δ^13^C was found in the biochar-amended soils relative to the corresponding controls over time (S3 Fig).

### Biochar carbon mineralisation and persistence in soil

The δ^13^C values of the respired CO_2_-C were consistently more depleted in the biochar-amended *vs.* non-amended soils, albeit not significantly on several occasions, with the depletion ranging from 0.3−3.1, 0.4−3.4, and 0.2−1.6‰ in the Arenosol, Cambisol and Ferralsol, respectively (S10[a] Fig). The proportion of biochar-C contribution to total C emission decreased with time, ranging between 2.2−22.5, 2.8−36.6, and 2.7−21.8% in the Arenosol, Cambisol and Ferralsol, respectively (S10[b] Fig).

### Biochar and Soil

The soil C/N ratio was significantly (*p ≤* 0.001) greater in the biochar-amended than the non-amended soils to 12 cm depth throughout the 12-month period. A relatively small but significant (*p ≤* 0.007) increase of soil C/N ratio was also observed in the 12−20 cm layer in the Ferralsol (S11 Fig).

**Temperature sensitivity of carbon emission rates**

The Q_10_ values of the biochar-C mineralisation and native SOC emissions (in the absence and presence of biochar) varied between 1.2 and 1.6 across soil types, compared to the results of controlled laboratory conditions [8, 9]. The confounding site-specific factors (e.g. soil properties and plant C input) across the contrasting field sites may have contributed to lowering the Q_10_ of biochar or native C emissions in the statistically similar range (S4 Table).

## References

1. Singh BP, Cowie AL, Smernik RJ. Biochar carbon stability in a clayey soil as a function of feedstock and pyrolysis temperature. Environ Sci Technol. 2012;46:11770-8. doi: 10.1021/es302545b.

2. Gillman G, Sumpter E. Modification to the compulsive exchange method for measuring exchange characteristics of soils. Soil Res. 1986;24(1):61-6.

3. MAFF. The analysis of agricultural materials. London: Ministry of Agriculture, Fisheries and Food, HMSO; 1986.

4. Baldock J, Hawke B, Sanderman J, Macdonald L. Predicting contents of carbon and its component fractions in Australian soils from diffuse reflectance mid-infrared spectra. Soil Res. 2014;51(8):577-95.

5. Kirschbaum MUF. The temperature dependence of soil organic matter decomposition, and the effect of global warming on soil organic C storage. Soil Biol Biochem. 1995;27(6):753-60. PubMed PMID: ISI:A1995QX82600003.

6. Singh N, Abiven S, Torn MS, Schmidt MWI. Fire-derived organic carbon turnover in soils on a centennial scale. Biogeosciences. 2012;9:2847-57.

7. Zimmerman AR. Abiotic and microbial oxidation of laboratory-produced black carbon (biochar). Environ Sci Technol. 2010;44(4):1295-301. doi: 10.1021/Es903140c. PubMed PMID: ISI:000274347800023.

8. Fang Y, Singh BP, Singh B. Temperature sensitivity of biochar and native carbon mineralisation in biochar-amended soils. Agric Ecosyst Environ. 2014;191:158-67.

9. Nguyen BT, Lehmann J, Hockaday WC, Joseph S, Masiello CA. Temperature sensitivity of black carbon decomposition and oxidation. Environ Sci Technol. 2010;44(9):3324-31. doi: 10.1021/Es903016y. PubMed PMID: ISI:000277067000023.

**Figure Captions**

**S1 Fig.** **Solid-state ^13^C cross polarisation (CP) Nuclear Magnetic Resonance (NMR) spectra of *Eucalyptus saligna* biochar.**

**S2 Fig.** **Soil bulk density (t m^-3^) of biochar-amended (black circle) and control (red empty circle) Arenosol, Cambisol and Ferralsol at 8 or 9 months and 12 months.** Error bars are standard errors (n = 4).

**S3 Fig 2.** **δ^13^C signature of biochar-amended and control Arenosol, Cambisol and Ferralsol.** The symbols of biochar-amended and non-amended (control) micro-plots are black circle and red empty circle, respectively. The data are presented at different depths and times after biochar incorporation in the soils. Error bars are standard errors (n = 4).

**S4 Fig.** **Above-ground biomass growth rate (g m^-2^ d^-1^) in Arenosol, Cambisol and Ferralsol during the experiment period (12 months).** There were no pasture swards in the Arenosol micro-plots in June, July and September 2013 as we used herbicides to eradicate *Digitaria* species infestation. The red symbol “_*_” represents the biochar has significant effect on plant growth. Error bars are standard errors (n = 4).

**S5 Fig. The correlation between changes in (Δlog10) total CO_2_-C emission rate and temperature and/or moisture in the biochar-amended (a) and control (b) micro-plots.** The R^2^ value was the squared correlation of the fitted values and the response. For calculating the p-value, a simple linear model was used.

**S6 Fig.** **The correlation between changes in (Δlog10) biochar-C mineralisation rate and temperature and/or moisture in the biochar-amended micro-plots.** The R^2^ value was the squared correlation of the fitted values and the response. For calculating the p-value, a simple linear model was used.

**S7 Fig. Cumulative amount of biochar-C mineralised and native C emission from plant and soil sources in biochar-amended and control Arenosol, Cambisol and Ferralsol over 12 months.** The symbols of biochar-amended and control are black circle and red empty circle, respectively. Error bars are standard errors (n = 4).

**S8 Fig. The proportion of applied biochar-C mineralised over 0−4 and 4−12 months.** Error bars are standard errors (n = 4).

**S9 Fig. The correlation of native soil C content with biochar-C mineralised (% of applied C).**

**S10 Fig. δ^13^C of soil-respired C (a, upper panel) and proportion (%) of biochar-derived C (b, lower panel) in soil plus biochar (total) CO_2_-C emitted from Arenosol, Cambisol and Ferralsol.** The symbols of biochar-amended and control (non-amended) micro-plots are black circle and red empty circle, respectively. Error bars are standard errors (n = 4).

**S11 Fig.** S**oil C/N ratio in biochar-amended and control Arenosol, Cambisol and Ferralsol.** The symbols of biochar-amended and control (non-amended) micro-plots are black circle and red empty circle, respectively. The data are presented at different depths and times after biochar incorporation in the soils. Error bars are standard errors (n = 4).

**S1 Fig**

**S2 Fig**

**S3 Fig**

**S4 Fig**

**S5 Fig**

**(a)**

**(b)**

**S6 Fig**

**S7 Fig**

**S8 Fig**

**S9 Fig**

**S10 Fig**

**S11 Fig**

**S1 Table. The estimated organic C composition (functional group proportion) of the woody biochar as determined by nuclear magnetic resonance (NMR) spectroscopy.**

|  | Alkyl | N-Alkyl/Methoxyl | O-Alkyl | Di-O-Alkyl | Aryl | O-Aryl | Amide/Carboxyl |
| --- | --- | --- | --- | --- | --- | --- | --- |
| Mixing model, % | 16 | 4.5 | 4.7 | 4.3 | 57.2 | 10.1 | 3.2 |

**S2 Table. The correlation co-efficient between temperature and moisture in biochar-amended and control soils.**

|  | **Arenosol** | **Cambisol** | **Ferralsol** |
| --- | --- | --- | --- |
| Biochar | -0.451 | -0.268 | 0.166 |
| Control | -0.404 | -0.231 | 0.071 |

**S3 Table.** **Microbial biomass (mg C kg^-1^ dry soil) at eight months in biochar-amended and control soils.** The values after ± are standard errors of means (n = 4).

|  | **Arenosol** | **Cambisol** | **Ferralsol** |
| --- | --- | --- | --- |
| Biochar | 122.0±7.2 | 298.7±21.9 | 619.4±52.2 |
| Control | 94.8±4.6 | 258.4±28.4 | 452.8±57.5 |

**S4 Table. Temperature sensitivity (Q_10_) of biochar and native soil organic carbon (SOC) in the absence and presence of biochar.**

|  | Biochar | Native SOC | |
| --- | --- | --- | --- |
|  |  | Control plots | Biochar plots |
| Arenosol | 1.4±0.4 | 1.4±0.2 | 1.4±0.2 |
| Cambisol | 1.6±0.6 | 1.4±0.4 | 1.6±0.5 |
| Ferralsol | 1.2±0.3 | 1.2±0.3 | 1.3±0.3 |
| The numbers after “±” are standard errors (n = 4). | | | |
